# Tratamento endovascular de aneurisma de tronco braquiocefálico em paciente com síndrome de Ehlers-Danlos tipo IV

**DOI:** 10.1590/1677-5449.001016

**Published:** 2016

**Authors:** Sergio Quilici Belczak, Rafael Kogan Klajner, Lara Cote Ogawa, Laís Leite Lucato, Bruna Stecca Zeque, Felipe Basso de Macedo, Ingredy Tavares da Silva, Luís Felipe Atihe

**Affiliations:** 1 Instituto Belczak de Cirurgia Vascular e Endovascular, São Paulo, SP, Brasil.; 2 Centro Universitário São Camilo – CUSC, São Paulo, SP, Brasil.

**Keywords:** aneurisma, tronco braquiocefálico, síndrome de Ehlers-Danlos

## Abstract

A síndrome de Ehlers-Danlos é uma doença genética que acarreta alteração na síntese de colágeno, causando extrema fragilidade do tecido conjuntivo. Tal fragilidade predispõe a uma série de doenças vasculares, como dissecções, aneurismas e pseudoaneurismas. Os autores relatam o histórico de um indivíduo de 19 anos com aneurisma de tronco braquiocefálico que foi submetido ao tratamento endovascular com implante de stents revestidos. O caso evoluiu com complicação do sítio de punção, que também foi tratada em caráter de emergência pela técnica endovascular com o implante de stent revestido.

## INTRODUÇÃO

A síndrome de Ehlers-Danlos é uma doença do tecido conjuntivo de caráter hereditário que se caracteriza por hipermobilidade, fragilidade e hiperextensibilidade da pele. É uma doença congênita cuja sintomatologia geralmente se exacerba no início da idade adulta. Atualmente, há 11 variantes já descritas, e cada uma delas apresenta características clínicas típicas, que variam de intensidade^1^.

A síndrome de Ehlers-Danlos tipo IV, ou síndrome de Sack-Barabas, é uma doença genética autossômica dominante que determina uma anomalia estrutural no colágeno de tipo III e provoca fragilidade em vasos sanguíneos, intestino, pulmões, pele, fígado, baço, etc. Há elevada possibilidade de rotura e dissecção arterial, mais frequentemente sem qualquer fator desencadeante. A rotura arterial espontânea é a principal causa de mortalidade entre os doentes que sofrem da síndrome (78,5%). Esse acidente arterial é raro durante a infância, mas 25% dos doentes sofrem um episódio inicial antes dos 20 anos e 80% antes dos 40 anos. Na Tabela 1, evidenciamos os critérios diagnósticos da síndrome de Ehlers-Danlos tipo IV^1^^-^ 4.

**Tabela 1:** Critérios maiores e menores para o diagnóstico de síndrome de Ehlers-Danlos tipo IV^2^.

**Síndrome de Ehlers-Danlos tipo IV**	
Defeito	Na produção de colágeno tipo III
Critérios maiores	Pele fina e transluscente^*^
	Fragilidade ou ruptura arterial/intestinal/uterina^*^ Aparência facial característica^*^ Hematomas frequentes e extensos^*^
Critérios menores	Acrogeria
	Hipermobilidade de pequenas articulações^*^
	Ruptura muscular e/ou tendínea
	Pé torto^*^
	Desenvolvimento precoce de varizes
	Fístula arteriovenosa (carótido-cavernosa)
	Pneumotórax^*^ História familiar positiva^*^
	Morte súbita

* Critérios apresentados pelo caso relatado.

Nesses pacientes, o seguimento inclui exame físico anual, ecocardiograma e ultrassonografia Doppler de carótidas e abdome. A arteriografia somente é indicada caso haja suspeita de complicações. Diversas complicações vasculares já foram relatadas. É comum o relato da presença de múltiplos aneurismas aórticos, periféricos e viscerais^5^^-^ 8.

## DESCRIÇÃO DO CASO

**Figura 1:**
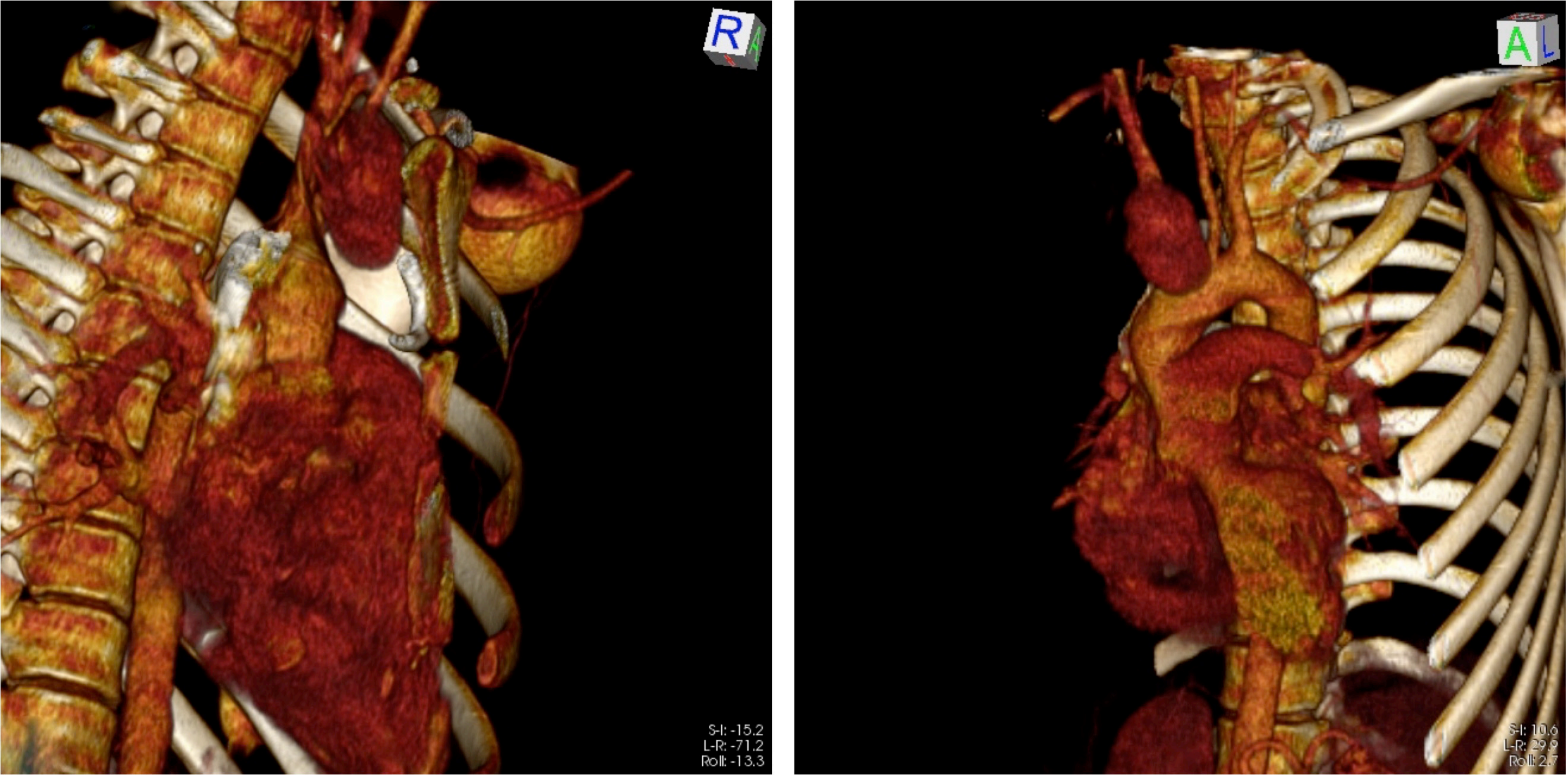
Imagens de reconstrução de angiotomografia evidenciando aneurisma de tronco braquiocefálico.

Paciente do sexo masculino, branco, 19 anos, com quadro de dor torácica aguda em hemitórax direito e dispneia moderada. Realizou radiografia de tórax que evidenciou tratar-se de quadro de pneumotórax espontâneo. Foi solicitada tomografia de tórax, que evidenciou grande massa em mediastino superior. Após a realização de angiotomografia, ela foi caracterizada como grande aneurisma de tronco braquiocefálico, sacular, medindo 7,2 cm em seu maior diâmetro (Figura 1).

O paciente já sabia do seu diagnóstico de síndrome de Ehlers-Danlos tipo IV. Relatou ter uma irmã com o mesmo diagnóstico e histórico recente de dissecção de carótida e de aorta abdominal tratadas clinicamente e sem sequelas.

**Figura 2:**
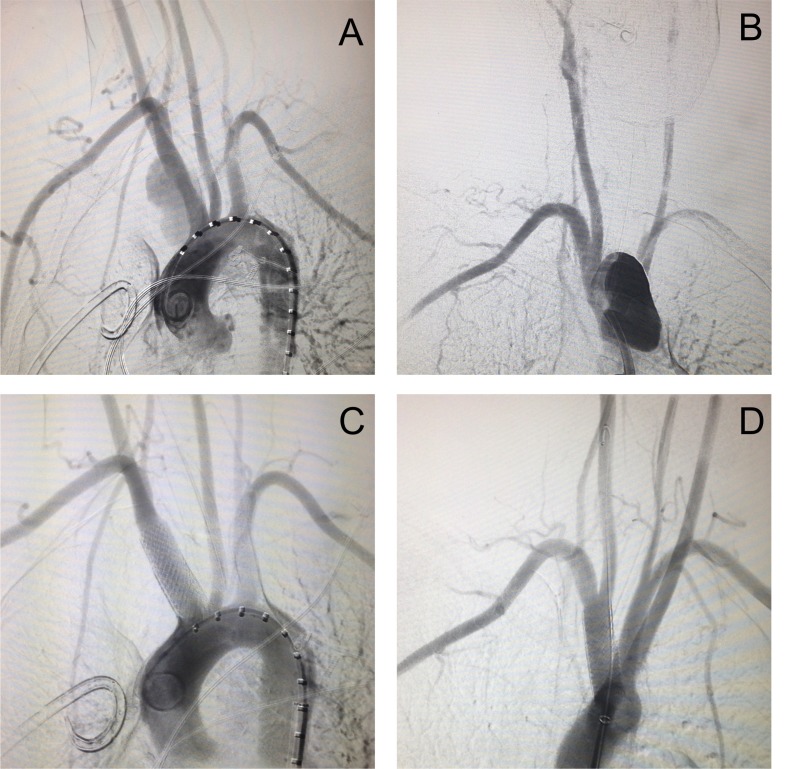
Imagens do tratamento endovascular do aneurisma de tronco braquiocefálico com stents revestidos. Arteriografia inicial (A, B); Arteriografia final (C, D).

Optou-se por correção endovascular do aneurisma. Por acesso femoral (9Fr), realizou-se implante de dois stents revestidos (Stent-graft Direct® 12x40), selando o colo do aneurisma e mantendo a perviedade das artérias carótida e subclávia, conforme evidenciado em angiografia de controle (Figura 2). Retirou-se o introdutor e realizou-se compressão manual do sítio de punção por 30 minutos, seguida da realização de curativo compressivo. O paciente foi encaminhado para a unidade de terapia intensiva sem queixas, assintomático e com parâmetros vitais estáveis.

**Figura 3:**
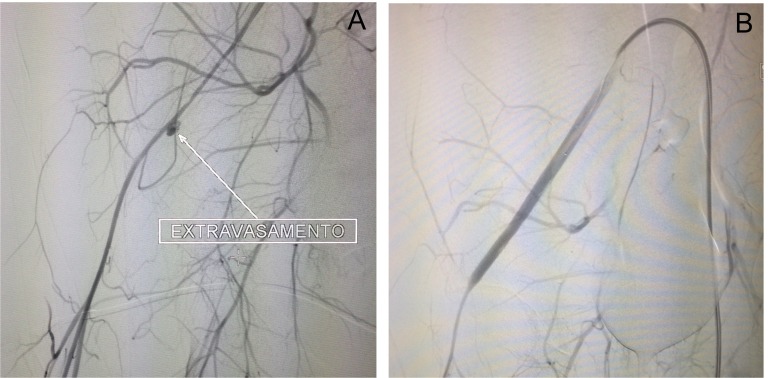
Imagens do tratamento endovascular do aneurisma de hemorragia do sítio de punção com stent revestido. Arteriografia inicial (A); Arteriografia final (B).

**Figura 4:**
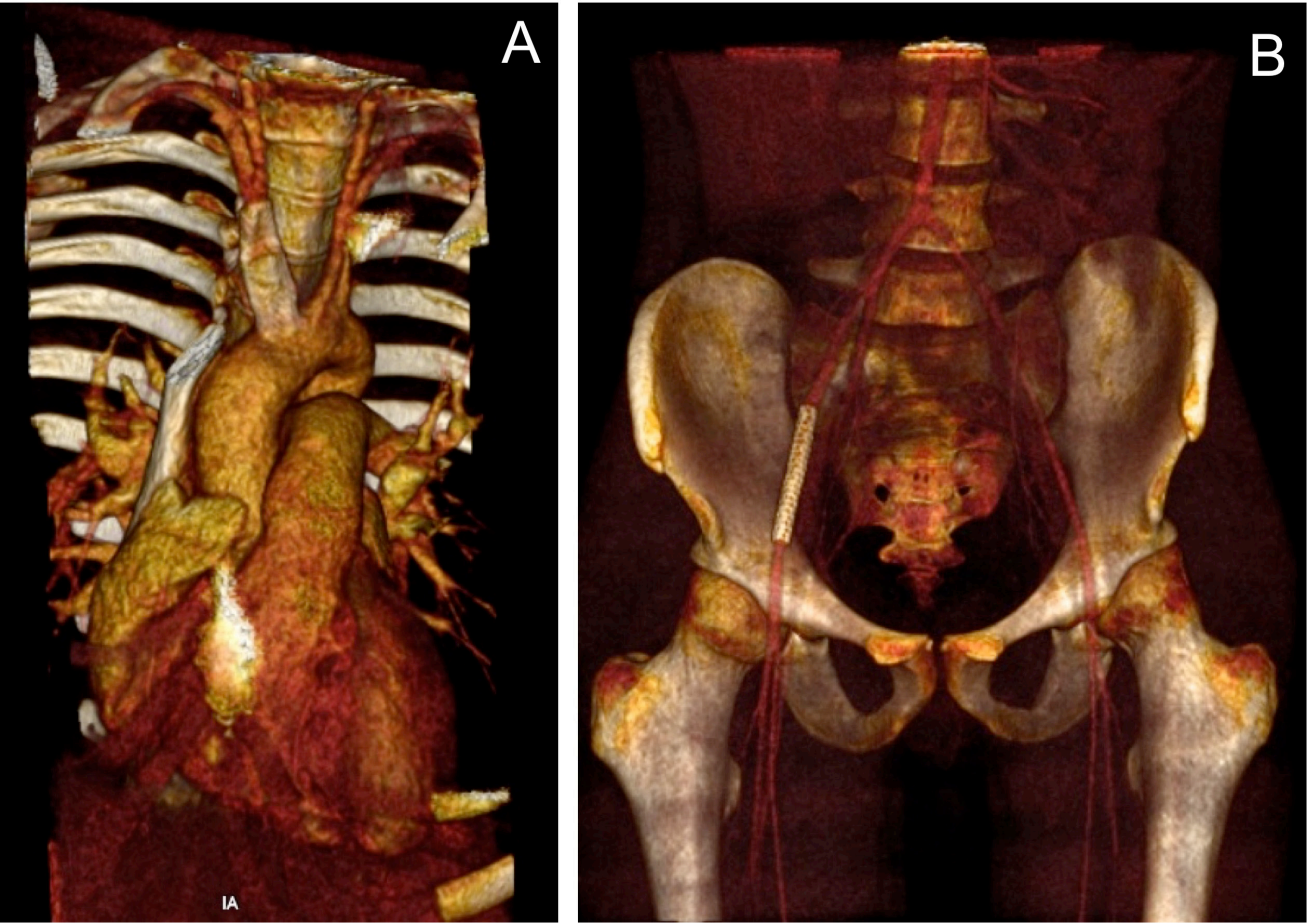
Imagens de reconstrução de angiotomografia de controle do tratamento de aneurisma de tronco braquiocefálico (A) e do sangramento da artéria ilíaca externa (B).

Uma hora após a realização do procedimento, o paciente evoluiu com choque hipovolêmico e foi levado em caráter emergencial novamente para hemodinâmica. Foi realizada nova angiografia, puncionando a artéria femoral contralateral. Ficou evidenciado sangramento posterior retroperitoneal na ilíaca externa próximo ao local de implante do introdutor no procedimento prévio. Nesse contexto, realizou-se implante de stent revestido (Advanta® 7x40), ocluindo o orifício do sangramento e mantendo a perviedade ilíaco-femoral (Figura 3). Implantou-se o dispositivo de selamento arterial Angioseal® (8Fr) no sítio de acesso femoral utilizado, seguido da realização de curativo compressivo. O paciente apresentou boa evolução e encontra-se em acompanhamento ambulatorial há 3 meses. Está assintomático, e a tomografia de controle evidenciou bom resultado (Figura 4).

## DISCUSSÃO

Foram relatadas inúmeras complicações vasculares decorrentes da fragilidade vascular nos pacientes portadores da síndrome de Ehlers-Danlos. Na Tabela 2, evidenciamos as complicações arteriais encontradas após uma revisão literária com 231 pacientes. Mais da metade desses pacientes tinham aneurismas, geralmente múltiplos e frequentemente em artérias viscerais. Porém, é necessário caracterizar também que há risco de ruptura arterial em vasos não aneurismáticos, espontânea ou decorrente de mínimos traumas^3^.

**Tabela 2:** Complicações vasculares em 231 pacientes portadores da síndrome de Ehlers-Danlos^3^.

**Complicações vasculares**	**Número de pacientes**	**Porcentagem de pacientes**
Aneurisma arterial^*^	93	40
Ruptura arterial (não aneurismática)	75	33
Fístula carótido-cavernosa	39	18
Grande hematoma (retroperitoneal/intestinal)	33	15
Hemorragia operatória^*^	30	13
Dissecção arterial	28	12
Pseudoaneurisma	21	9
Ruptura aneurismática	23	10
Fístula arteriovenosa	5	2
Aneurisma de coronária	2	1

* Complicações da doença apresentadas pelo caso relatado.

A indicação de tratamento desses pacientes deve ser ponderada frente ao elevado risco de complicações. Há elevada incidência de complicações vasculares, incluindo dissecções arteriais e rupturas no sítio de acesso^2^. Em revisão da Mayo Clinic, caracterizou-se uma mortalidade de 46% após o tratamento aberto ou endovascular de pacientes portadores da síndrome^5^.

Na Tabela 3, evidenciamos os tratamentos e a mortalidade pós-cirúrgica de 119 pacientes após revisão literária. Esses tratamentos incluem inúmeros tipos de reconstruções vasculares abertas, ligaduras, exclusões de órgãos (baço e rim), entre outros. O tratamento endovascular foi realizado em 32 pacientes. O grupo miscelâneas é formado por uma variedade de procedimentos, como pacientes submetidos a exérese de órgão que evoluíram a óbito antes do tratamento ou cujo diagnóstico foi realizado durante uma autópsia.

**Tabela 3:** Procedimentos cirúrgicos realizados e mortalidade em curto prazo (em 30 dias após o procedimento) em 119 pacientes portadores de síndrome de Ehlers-Danlos^3^.

	**Cirurgia aberta**	**Cirurgia endovascular** ^*^	**Miscelânea**	**Total**
Tratamento (n)	44	33	42	119
Mortalidade (n/%)	13/30	8/24	25/60	46/39

* Procedimento cirúrgico realizado no caso relatado.

Houve uma diferença mínima entre o grupo de cirurgia aberta e o de cirurgia endovascular. O grupo com menor taxa de complicações foi o dos pacientes submetidos a embolização (20% - cinco de 25 casos), e o grupo com maior taxa foi o de miscelâneas (60%).

Assim, na revisão literária, da mesma forma que no caso apresentado, ficou evidente uma incidência elevada de complicações após o tratamento cirúrgico dos pacientes. Depois do tratamento endovascular, dois pacientes morreram de acidente vascular cerebral (AVC) após o implante de stents ou embolização com molas de fístulas carótido-cavernosas; um paciente morreu de AVC após o implante de endoprótese torácica; um paciente morreu de ruptura da artéria ilíaca após implante de stent em artéria vertebral; e um paciente morreu de sangramento após embolização mesentérica. Todos os pacientes no grupo de miscelâneas faleceram de complicações hemorrágicas. A média de idade dos pacientes que foram a óbito foi de 31 anos (de 5 meses até 53 anos)^2^^-^ 4.

## CONCLUSÃO

A partir desses dados, concluímos que o prognóstico é ruim após o tratamento, seja ele aberto ou endovascular, com uma taxa de mortalidade de 29% na revisão de literatura. Frente à raridade da doença e à pequena quantidade de publicações, sugere-se a realização de um registro nacional e internacional para coleta de dados e futuras publicações.
