# Supplementary material for: Tratamento endovascular de aneurisma de tronco braquiocefálico em paciente com síndrome de Ehlers-Danlos tipo IV
Source: J Vasc Bras. 2016 Jul-Sep;15(3):239–44. [Article in Portuguese] doi: 10.1590/1677-5449.001016 (PMC5829762; doi:10.1590/1677-5449.001016)
Supplement: Translation

# Translation: Endovascular treatment of an aneurysm of the brachiocephalic trunk in a patient with Ehlers-Danlos syndrome type IV

**Authors:** Sergio Quilici Belczak, Rafael Kogan Klajner, Lara Cote Ogawa, Laís Leite Lucato, Bruna Stecca Zeque, Felipe Basso de Macedo, Ingredy Tavares da Silva, Luís Felipe Atihe

**Affiliations:** 1 Instituto Belczak de Cirurgia Vascular e Endovascular, São Paulo, SP, Brazil.; 2 Centro Universitário São Camilo – CUSC, São Paulo, SP, Brazil.

**Keywords:** aneurysm, brachiocephalic trunk, Ehlers-Danlos syndrome

## Abstract

Ehlers-Danlos syndrome is a genetic disease that results in abnormalities of collagen synthesis, causing extremely fragile connective tissue. This fragility predisposes patients to a series of vascular disorders, such as dissections, aneurysms and pseudoaneurysms. The authors describe the case of a 19-year-old patient with an aneurysm of the brachiocephalic trunk who was treated endovascularly by placement of covered stents. The case progressed with complications at the puncture site, which was also treated on an emergency basis, using endovascular techniques with a covered stent.

## INTRODUCTION

Ehlers-Danlos syndrome is a hereditary connective tissue disease that is characterized by hypermobility, fragility and hyperextensibility of the skin. It is a congenital disease and symptomology generally exacerbates at the start of adulthood. To date, 11 variants have been described and each one exhibits typical clinical characteristics that vary in intensity.^1^

Ehlers-Danlos syndrome type IV, also known as Sack-Barabas syndrome, is a dominant autosomal genetic disease that results in a structural anomaly of type III collagen and causes fragility in blood vessels, intestines, lungs, skin, liver, and spleen, etc. There is a high likelihood of arterial rupture and dissection, very often with no trigger factor whatsoever. Spontaneous arterial rupture is the primary cause of mortality among patients who have the syndrome (78.5%). This arterial accident is rare during childhood, but 25% of patients will suffer an initial episode before the age of 20 and 80% before they reach 40. Table 1 lists the diagnostic criteria for Ehlers-Danlos syndrome type IV.^1^^-^ 4

**Table 1:** Major and minor criteria for diagnosis of Ehlers-Danlos syndrome type IV^2^.

**Ehlers-Danlos syndrome type IV**	
Defect	Of type III collagen production
Major criteria	Thin and translucent skin^*^
	Arterial/intestinal/uterine fragility or rupture^*^ Characteristic facial appearance^*^ Frequent and extensive hematomas^*^
Minor criteria	Acrogeria
	Hypermobility of small joints^*^
	Rupture of muscles and/or tendons
	Twisted feet^*^
	Premature development of varicose veins
	Arteriovenous fistula (carotid cavernous)
	Pneumothorax^*^ Positive family history^*^
	Sudden death

* Criteria observed in the case reported here

Management of these patients includes an annual physical examination, echocardiogram and Doppler ultrasonography of the carotids and abdomen. Arteriography is only indicated if there is a suspicion of complications. Several vascular complications have been reported. Reports of multiple aortic, peripheral and visceral aneurysms are common.^5^^-^ 8

## CASE DESCRIPTION

A 19-year-old, white, male patient presented with acute chest pain in the right hemithorax and moderate dyspnea. A chest X-ray showed that he was suffering from spontaneous pneumothorax. A tomography of the chest was conducted, which showed a large mass in the upper mediastinum. After angiotomography had been performed, this was identified as a large, saccular aneurysm of the brachiocephalic trunk with a maximum diameter of 7.2 cm (Figure 1).

**Figure 1:** Reconstructed angiotomography images showing an aneurysm of the brachiocephalic trunk.

The patient had already been diagnosed with Ehlers-Danlos syndrome type IV and was aware of this. He reported having a sister with the same diagnosis who had recently suffered dissection of the carotid and abdominal aorta, which had been treated clinically without sequelae.

The decision was taken to attempt an endovascular aneurysm repair. A femoral access (9Fr) was used to place two covered stents (12x40 Direct Stent-graft®), sealing the aneurysm while preserving patency of the carotid and subclavian arteries, which was confirmed by a control angiography (Figure 2). The introducer was withdrawn and manual compression was applied to the puncture site for 30 minutes, followed by a pressure dressing. The patient was transported to the intensive care unit free from complaints, asymptomatic and with stable vital signs.

**Figure 2:** Images of endovascular treatment to repair an aneurysm of the brachiocephalic trunk with covered stents. Initial arteriography (A, B); Final arteriography (C, D).

An hour after the procedure, the patient developed hypovolemic shock and was taken back to the hemodynamics suite for emergency treatment. Angiography was performed once more, via puncture of the contralateral femoral artery. Retroperitoneal bleeding was detected from the posterior external iliac close to the site where the introducer had been used in the previous procedure. In response to this, a covered stent (Advanta® 7x40) was implanted to occlude the bleeding orifice while maintaining iliofemoral patency (Figure 3). An Angioseal® (8Fr) arterial sealing device was implanted at the site of femoral access, followed by a pressure dressing. The patient recovered well and at the time of writing he had been in outpatients follow-up for 3 months and was asymptomatic, and a control tomography showed good results (Figure 4).

**Figure 3:** Images of endovascular treatment of hemorrhaging aneurysm at the puncture site using a covered stent. Initial arteriography (A); Final arteriography (B).

**Figure 4:** Reconstructed images from control angiotomography after treatment to repair aneurysm of the brachiocephalic trunk (A) and external iliac bleeding (B).

## DISCUSSION

There are countless reports of vascular complications resulting from vascular fragility in patients with Ehlers-Danlos syndrome. Table 2 lists the arterial complications described in studies of a total of 231 patients that were identified in a literature review. More than half of these patients had aneurysms, generally multiple and frequently in visceral arteries. However, it should be noted that there is also a risk of arterial rupture in vessels that do not have aneurysms and these can be spontaneous or caused by minimal traumas.^3^

**Table 2:** Vascular complications in 231 patients with Ehlers-Danlos syndrome^3^.

**Vascular complications**	**Number of patients**	**Percentage of patients**
Arterial aneurysm^*^	93	40
Arterial rupture (non-aneurysmal)	75	33
Carotid cavernous fistula	39	18
Major hematoma (retroperitoneal/intestinal)	33	15
Operative hemorrhage^*^	30	13
Arterial dissection	28	12
Pseudoaneurysm	21	9
Aneurysmal rupture	23	10
Arteriovenous fistula	5	2
Coronary aneurysm	2	1

* Complications of the disease observed in the case reported here.

In these patients, treatment indications should be weighed up against the high risk of complications. There is an elevated incidence of vascular complications, including arterial dissections and ruptures at the access site.^2^ A review conducted by the Mayo Clinic identified a 46% mortality rate after open or endovascular treatment of patients with this syndrome.^5^

Table 3 lists treatment types and post-surgical mortality in 119 patients compiled after a literature review. These treatments included countless types of open vascular reconstruction, ligations, exclusion of organs (spleen and kidney), and other procedures. Endovascular techniques were used to treat 32 patients. The miscellaneous group encompasses a variety of procedures, such as patients subjected to exeresis of organs who died before treatment or who were diagnosed during autopsies.

**Table 3:** Surgical procedures performed and short-term mortality (within 30 days of procedure) in 119 patients with Ehlers-Danlos syndrome^3^.

	**Open surgery**	**Endovascular surgery** ^*^	**Miscellaneous**	**Total**
Treatment (n)	44	33	42	119
Mortality (n/%)	13/30	8/24	25/60	46/39

* Surgical procedure performed in the case reported here.

There was a minimal difference between the open surgery and endovascular surgery groups. The group with the lowest rate of complications was a subset of patients who underwent embolization (20% - five out of 25 cases), and the group with the highest rate was the miscellaneous category (60%).

As these figures show, both the literature review and the case report make it clear that there is an elevated incidence of complications after surgical treatment of these patients. After endovascular treatments, two patients died from cerebral vascular accidents after stent placement or coil embolization for carotid-cavernous fistulas; one patient died from a cerebral vascular accident after placement of a thoracic endoprosthesis; one patient died from a ruptured iliac artery after stent placement in the vertebral artery; and one patient died from bleeding after mesenteric embolization. All of the patients in the miscellaneous category died from hemorrhagic complications. The mean of age of patients who died was 31 years (range: 5 months to 53 years).^2^^-^ 4

## CONCLUSIONS

On the basis of these data it is concluded that prognosis is poor after treatment, whether open or endovascular, with a 29% mortality rate in the literature review. In view of the rarity of this disease and the low number of publications on the subject, it is suggested that national and international registers should be set up to enable data collection and provide a basis for future publications.
